# Is There a Role for Dose Modification of TKI Therapy in CML?

**DOI:** 10.1007/s11899-019-00524-w

**Published:** 2019-06-14

**Authors:** M. Copland

**Affiliations:** Institute of Cancer Sciences, College of Medical, Veterinary and Life Sciences, Gartnavel General Hospital, University of Glasgow, Paul O’Gorman Leukaemia Research Centre, 1053 Great Western Road, Glasgow, G12 0YN Scotland

**Keywords:** Chronic myeloid leukemia, Tyrosine kinase inhibitors, Dose optimization, Treatment-free remission, Toxicity, Elderly

## Abstract

**Purpose of Review:**

For patients with chronic phase chronic myeloid leukemia (CP-CML), there is an increasing focus on personalization of therapy with dose modifications of tyrosine kinase inhibitors (TKIs) to reduce side effects and maintain efficacy. Dose reductions are also being considered in clinical trials prior to treatment-free remission (TFR) attempts.

**Recent Findings:**

Recent retrospective analyses of large clinical trials show that dose modification/reduction is safe. Efficacy is generally maintained and side effects are improved. Clinical trials such as DESTINY have demonstrated that dose reduction is safe for patients in deep molecular remission and may be considered prior to a TFR attempt.

**Summary:**

Dose modifications are widely used to prevent and manage the toxicities of TKIs. With adequate monitoring, dose optimization is safe, reduces side effects, and improves quality-of-life for patients. Clinical trials of dose optimization are currently recruiting across all approved TKIs and will lead to further personalization of therapy for CP-CML patients in the future.

## Introduction

The introduction of the tyrosine kinase inhibitors (TKI) imatinib, dasatinib, nilotinib, bosutinib, and ponatinib has revolutionized the treatment of chronic myeloid leukemia (CML). The vast majority of patients with CML (> 85%) are diagnosed in chronic phase (CP); however, if untreated, CP-CML will progress through an accelerated phase to a fatal acute leukemia, termed blast crisis, within 5–7 years.

Recent clinical trial data from the German CML IV study confirm that in excess of 80% of CP-CML patients are expected to be alive after 10 years of TKI therapy, with a 10-year relative survival of 92% compared to the general population [1]. Many of these patients will have near normal life expectancy with no progression to advanced phase disease. A recent study from the Swedish Cancer Registry showed that patients diagnosed in 2013 lose, on average, three life years to CML [2]. Thus, the prevalence of CML is rapidly increasing and, in the future, will become the most common form of leukemia seen in the outpatient clinic. Further data from the Swedish Cancer Registry shows that the prevalence of CML has tripled between 1985 and 2012 from 3.9 to 11.9 per 100,000 population, and there is a projected further increase in CML prevalence to 22 per 100,000 inhabitants by 2060 [3]. Similar results were obtained in a recent French study by Delord et al. [4]. Both studies highlight the rising economic and social burdens of CML, with expanding costs for medication and disease monitoring, together with the side effects of TKIs affecting quality-of-life for patients, and potentially causing more serious health issues. Both the economic burden and the side effect profile of the TKIs highlight the importance of taking a personalized approach to dose modification for CML patients.

### Monitoring Response to Therapy in CML

For patients established on a TKI, therapy is monitored using quantitative (q)RT-PCR to measure the amount of *BCR-ABL*, the causative fusion oncogene of CML [5]. QRT-PCR for *BCR-ABL*, compared to a housekeeping gene (usually *ABL*), accurately reflects the amount of residual leukemia and, at time points from 3 months of therapy, is used to determine whether patients have an optimal response to TKI. This is defined as achievement of ≦ 10% *BCR-ABL*:*ABL* transcripts at 3 months (termed early molecular response (EMR)), [6, 7] and major molecular response (MMR) (*BCR-ABL*:*ABL* transcripts < 0.1% on the international scale (IS)] from 12 months of TKI therapy onwards [8]. Once MMR is achieved and maintained, the risk of progression to advanced phase CML is extremely low [9]. Furthermore, a proportion of patients on prolonged TKI therapy will become persistently *BCR-ABL* negative, providing the tantalizing hope that TKIs may be curing some CML patients. Indeed, trials of treatment-free remission (TFR) are showing very encouraging results, with a proportion of optimally responding patients being able to successfully discontinue TKI therapy (reviewed in references [10, 11]).

### Are We Overtreating Optimally Responding Patients?

For optimally responding patients, we need to consider whether or not they are being overtreated with standard doses of TKI, or indeed, if they continue to require therapy for their CP-CML at all. This is a significant issue, as while the TKIs and imatinib in particular have an excellent safety profile, many patients experience persistent low-level side effects which impact on quality-of-life [12]. In addition, there is increasing evidence for more serious side effects with second generation (2G-)TKIs which have the potential to cause significant morbidity and mortality in some patients, for example, pleural effusion and pulmonary arterial hypertension with dasatinib [13]; dyslipidemia and arterial thrombotic events with nilotinib [14, 15]; diarrhea and liver dysfunction with bosutinib [16]; and hypertension, arterial thrombotic events, and liver dysfunction with ponatinib [17•].

Dose modifications of TKIs in CML must have two complimentary aims—the achievement and maintenance of cytogenetic and molecular responses, together with a reduction in side effects. This review will discuss the evidence for dose modifications throughout the CML patient’s treatment journey. It will consider dose modifications for newly diagnosed patients, including the elderly, dose reduction during therapy, often for side effects, and dose reduction for patients in deep molecular remission (DMR) (defined as *BCR-ABL*:*ABL* ratio of < 0.01%^IS^) [18], either as a prelude to an attempt at TFR or as continuous maintenance therapy in those patients not wishing to attempt TFR.

### Dose Modifications in Patients with Newly Diagnosed CP-CML

It is widely accepted that the standard starting dose of imatinib is 400 mg/day. In clinical trials, doses above 400 mg/day have resulted in superior efficacy, but at the expense of a worse side effect profile, with consequent effects on quality-of-life [1, 19]. There is very limited data for starting imatinib at a dose below 400 mg daily, unless for very elderly patients or those with significant comorbidities and polypharmacy where side effects and drug interactions are a concern [20•].

The Optimized Tyrosine Kinase Inhibitor Monotherapy (OPTIM)-imatinib clinical trial was a randomized study in patients with newly diagnosed CP-CML to consider the utility of dose optimization based on minimal (trough) plasma concentrations [C]_min_ of imatinib 2 weeks after enrolment into the trial [21]. Those patients with a [C]_min_ < 1000 ng/mL were randomized to either a dose increase strategy to obtain [C]_min_ ≥ 1000 ng/mL on continue or 400 mg/day. Those patients with a [C]_min_ ≥ 1000 ng/mL at the first assessment remained on imatinib 400 mg daily. The primary endpoint was MMR at 12 months. The MMR rate in the standard imatinib arms was 37%, which improved to 63% in the dose-optimization arm. There was no significant difference in MMR rate between the dose-optimization arm and those with [C]_min_ ≥ 1000 ng/mL at the first assessment. Interestingly, only one-third of newly diagnosed CP-CML patients on imatinib 400 mg daily had [C]_min_ ≥ 1000 ng/mL, supporting a role for personalized treatment strategies and dose optimization for patients commencing imatinib.

The Therapeutic Intensification in De Novo Leukemia (TIDEL I and II) clinical trials evaluated commencing with a starting dose of imatinib of 600 mg daily and intensifying therapy for suboptimal response. TIDEL 1 demonstrated superior responses in those patients able to tolerate 600 mg daily, indicating that early dose intensity may be critical for optimizing response [22]. TIDEL II was a randomized study in which all patients were commenced on imatinib 600 mg daily. Patients with a suboptimal response from 3 months onwards were randomized to either dose escalation to 800 mg daily followed by switch to nilotinib for continued suboptimal response versus switching directly to nilotinib [23]. With an overall survival of 96% and progression-free survival of 95% at 3 years, this trial demonstrated the feasibility of initial therapy with imatinib with an early switch to nilotinib for lack of response.

In many clinical trials of 2G-TKIs, it was quickly discovered that lower doses were as efficacious, with a better safety profile than the maximum tolerated doses achieved in early phase clinical trials (see Table 1). For example, the CA180-034 study, in imatinib-resistant and intolerant patients, demonstrated that 100-mg dasatinib once daily was as effective as both 140 mg once daily or 70 mg twice daily with a more favorable toxicity profile [24•]. This was confirmed in the DASISION clinical trial [25] in newly diagnosed CP-CML, where the superior efficacy to imatinib and improved safety compared to higher doses of dasatinib used in previous trials [30] were maintained over many years.

**Table 1:** Seminal dose-optimization studies of dasatinib and nilotinib

TKI	Setting	Doses	% MMR at any time	Accepted TKI dose	Reference
Dasatinib CA180-034 study	Resistance/intolerance	50 mg bd 100 mg od 70 mg bd 140 mg od	44% 46% 44% 46%	100 mg od for all chronic phase indications	[24•]
Dasatinib DASISION	Newly diagnosed	100 mg od versus imatinib 400 mg od	76% 64%		[25]
Nilotinib ENESTnd study	Newly diagnosed	300 mg bd 400 mg bd versus imatinib 400 mg od	77% 77% 60%	300 mg bd in newly diagnosed and 400 mg bd for resistance/intolerance	[26]
Bosutinib BELA	Newly diagnosed	500 mg versus imatinib 400 mg od	59% at 24 months 49% at 24 months	400 mg od in newly diagnosed and 500 mg od for resistance/intolerance	[27]
Bosutinib BFORE	Newly diagnosed	400 mg versus imatinib 400 mg od	61.2% at 24 months 50.7% at 24 months		[28•, 29]

A similar picture is also seen with nilotinib, where the ENESTnd study demonstrated the equivalent efficacy of nilotinib 300 mg and 400 mg twice daily [26], both of which were superior to imatinib 400 mg daily. Interestingly, nilotinib 400 mg bd, but not nilotinib 300 mg bd, showed superior 5-year overall survival compared to imatinib (*P* = 0.0266; imatinib 91.7%, nilotinib 300 mg bd 93.7%, and 400 mg bd 96.2%). However, this was countered by a higher rate of cardiovascular events in the nilotinib 400 mg bd arm (cardiovascular events of any grade: imatinib 2.1%, nilotinib 300 mg bd 7.5%, and 400 mg bd 13.4%). Based on this, the recommended dose for newly diagnosed patients is 300 mg twice daily and 400 mg twice daily for second-line therapy [31].

BELA, the original first-line study of bosutinib in newly diagnosed CP-CML failed its primary endpoint of demonstrating superior cytogenetic response to imatinib, in part due to the side effect profile of bosutinib at the recommended dose of 500 mg daily [16]. In a subsequent study (BFORE), using a lower dose of 400 mg daily in newly diagnosed patients, superiority to imatinib was observed [28•], again with an improved side effect profile.

In recent years, with our increased knowledge of the potential of TKIs to cause significant morbidity and mortality, together with their accepted effects in reducing quality-of-life, there has been a renewed focus on dose modifications in an attempt to reduce side effects, minimize treatment interruptions, and obtain optimal molecular responses for patients.

As with OPTIM-imatinib, the phase 2 OPTIM-dasatinib study used therapeutic drug monitoring (TDM) as a basis for dose optimization [32, 33]. Patients with a high [C]_min_ of ≥ 3/nmol/L at the first assessment had dasatinib dose reduced by 20 mg every 2 weeks to a minimum dosage of 40 mg/day to obtain a plasma [C]_min_ of < 3/nmol/L. Patients randomized to the TDM strategy had a reduced risk of pleural effusion (11% versus 45% by 36 months, *P* = 0.008) and discontinuation (13% versus 27%) compared to the control group. At 12 months, the MMR rates were equivalent between the arms.

Very recently, Naqvi et al. reported early results of a single-arm phase 2 study exploring the efficacy and safety profile of dasatinib 50 mg daily in newly diagnosed CP-CML [34••]. The primary endpoint was MMR rate at 12 months. Seventy-five patients were recruited with a median age of 47 years (range 19–84). At the time of reporting, 60 patients were evaluable for 3-month response, with 56/60 patients (93%) and 43/60 (72%) achieving *BCR-ABL*:*ABL*^*IS*^ of ≤ 10% and ≤ 1%, respectively. Twenty-four patients had 12-months follow-up, and of these, 19/24 (79%) were in MMR, and 11/24 (71%) were in DMR. There were no transformations to advanced phase, and only one reported case of pleural effusion (grade 1). Nine patients had dose interruptions of < 14 days for non-hematologic adverse events (*N* = 6) or thrombocytopenia (*N* = 3). Although the results are very preliminary, compared to the historical DASISION study [35], response rates at early time points are higher in this study, and a phase 3 comparison of dasatinib 50 mg versus 100 mg may now be warranted.

### Dose Modifications for Patients Established on TKI Therapy for Management of Side Effects

The majority of TKI dose modifications are dose reductions, instituted for the management of TKI-related side effects [20•]. There are multiple published case reports and case series of patients reducing imatinib dose due to intolerance, and achieving and maintaining molecular response on imatinib doses below the standard dose of 400 mg daily. For example, Carella and Lerma [36] published a series of 4 patients, intolerant to standard dose imatinib, who remained *BCR-ABL* negative for a median of 17 months (range 4–37 months) on 200 mg daily.

In the Italian single-arm phase 2 INTERIM study [37], a different approach was adopted, and Russo et al. reported outcomes for 76 elderly patients (aged 65–83 years) treated with an intermittent imatinib schedule (alternate months on and off imatinib). Enrolled patients were in complete cytogenetic remission (CCyR) and MMR, and had been on imatinib for at least 2 years. With a minimum of 4 years follow-up, 27 and 13 patients had lost MMR and CCyR, respectively. All patients losing response resumed continuous TKI therapy, and 26/27 re-achieved CCyR and MMR, with one patient lost to follow-up. There were no progressions to advanced phase.

Dose modifications are much more common with 2G- and 3G-TKIs. In a retrospective analysis of the DASISION clinical trial, dasatinib dose reductions did not affect efficacy, and the superior MMR rate of dasatinib was maintained [38]. In the NORDCML006 study [39], comparing imatinib 400 mg/day with dasatinib 100 mg/day in newly diagnosed CP-CML patients (*n* = 46), dasatinib dose reductions for adverse events were frequent; however, molecular responses were maintained. Six patients dose reduced dasatinib to a mean of 50 mg/day, and an additional 3 patients switched from imatinib to low-dose dasatinib. After 36 months, only 36% of patients randomized remained on full-dose dasatinib, a further 27% were on a reduced dose (mainly due to toxicity). However, molecular response rates were equivalent between the standard and reduced doses.

The Japanese LD-CML study switched patients on imatinib doses of ≤ 200 mg daily to dasatinib 50 mg daily [40]. Of the nine patients recruited (median age 73 years, range 64–87), eight were not in MMR. Five of eight achieved the primary endpoint of MMR at 12 months and 3/9 achieved DMR. There were no dasatinib dose escalations, five patients required dose interruption, and two required dose reduction for management of side effects. There were no transformations to advanced phase and no treatment discontinuations for the 18-month duration of the study.

The DARIA 01 study was a phase 2 study exploring factors which influenced adherence and efficacy [41], and correlated dasatinib [C]_min_ on day 28 of therapy with likelihood of dose reduction. Twenty-eight and twenty-five percent of patients, respectively, had a dose reduction or dose interruption. Overall, 34% of patients had their dasatinib dose reduced. In multivariate analysis, high [C]_min_, older age, and poor performance status correlated with dose reduction.

In addition to reducing from twice daily to once daily dosing of dasatinib, an intermittent treatment schedule for dasatinib has been reported in patients with resistance/intolerance to imatinib, with the aim of reducing toxicity and maintaining efficacy. In a retrospective German study, 33 patients were treated with an “on-off” dasatinib regimen (3–5 days on therapy followed by 2–4 days off therapy) in an attempt to reduce dasatinib-related toxicity [42]. Pleural effusions and hematological toxicity were significantly reduced, and efficacy of this strategy was demonstrated in 58% of evaluable patients. To more fully evaluate this strategy of dose optimization, the Dasatinib Holiday for Improved Tolerability (DasaHIT) clinical trial is currently randomizing both newly diagnosed CP-CML patients and patients with resistance or intolerance to alternative TKIs to dasatinib 100 mg/day continuously versus dasatinib 100 mg for 5 of 7 days (NCT02890784).

Other than the published ENESTnd clinical trial [26], there are few studies exploring dose optimization with nilotinib. In a single-center study, Santos et al. assessed the impact of dose reductions and interruptions of nilotinib and dasatinib in 280 patients across all phases of CML [43]. In total, 129 patients received nilotinib and 151 received dasatinib. Dose reductions/interruptions were more frequent with dasatinib than nilotinib, with 113 (75%) of patients on dasatinib and 63 (49%) patients on nilotinib requiring a dose reduction (*P* < 0.0001). Using multivariate regression analysis, older age, female sex, and use of dasatinib were independently associated with dose modification. Importantly, there was no significant difference in efficacy between those patients that did or did not have a dose reduction with either nilotinib or dasatinib.

The NILO-RED observational study recruited 81 patients to a more convenient once daily dosing regimen of nilotinib 300–450 mg [44•]. At the time of reporting, the first 67 patients were evaluable with a minimum follow-up of 12 months. The study included patients on both first-line nilotinib (*n* = 46, 300 mg twice daily) and second-line nilotinib (*n* = 21, 400 mg twice daily). Two of the 46 first-line patients and none of the second-line patients lost MMR after nilotinib dose reduction; in these two patients, MMR was spontaneously regained 4 and 6 months later. All patients dose reducing in DMR and 8 of 10 patients reducing in MMR maintained response at 12 months. In addition, a subset of these patients went on to attempt TFR. The NILO-RED study provides preliminary evidence that a switch to nilotinib maintenance at a once daily dose is feasible and safe, regardless of prior therapies. Based on these results, further prospective clinical trials are now required to more fully evaluate nilotinib dose optimization schedules incorporating once daily dosing in optimally responding patients.

In a phase 1/2 trial of bosutinib for patients with all phases of CML, failing two or more prior TKIs, dose reductions to 400 mg or 300 mg per day or treatment interruptions were required in 45% and 65% of patients, respectively [45]. In the BFORE study in newly diagnosed CP-CML patients, adverse events were successfully managed with bosutinib dose reductions to 200 mg or 300 mg daily in 103 patients [46]. Twenty of 103 patients were in MMR at time of dose reduction, and only one patient lost MMR after dose reduction. An additional 40 patients obtained MMR after dose reduction. In an ongoing bosutinib dose-optimization study at the MD Anderson Cancer Center, in patients that have failed their first-line TKI, bosutinib is being commenced at a dose of 300 mg per day, and then increased if required in an attempt to minimize side effects and improve tolerability (NCT02906696). These studies highlight that dose optimization for bosutinib continues.

Moving on to consider dose optimization for the 3G-TKI ponatinib, a phase 2 dose-finding study, OPTIC (NCT02467270), has very recently completed recruitment to establish the most effective dose of ponatinib together with minimization of side effects, in particular, arteriothrombotic events (ATE). The approval for ponatinib at a dose of 45 mg daily was based on the PACE clinical trial, published in 2013 which demonstrated the efficacy of ponatinib in patients with resistance and intolerance to multiple TKIs across all phases of disease, including those with T315I mutations [47]. However, longer follow-up of PACE identified a high and increasing rate of ATE in patients treated with ponatinib which raised significant safety concerns [17•], led to immediate dose reductions to 15 mg in responding patients, the abandonment of the first-line EPIC clinical trial comparing ponatinib 45 mg daily with imatinib [48], and led to an FDA black box warning for ponatinib. Modeling the data from PACE suggested that there was a dose-effect relationship for both response and side effects. Thus, lowering the dose may reduce cardiovascular toxicity but maintain efficacy. This hypothesis is being tested in OPTIC to determine the optimal starting dose of ponatinib. Patients with resistance to at least two other TKIs are being randomized between doses of 15-mg, 30-mg, and 45-mg ponatinib. On achievement of major cytogenetic remission (MCyR) or *BCR-ABL*:*ABL*^IS^ ratio ≤ 1%, the dose is reduced to 15 mg for all patients. The primary endpoint is MCyR by 12 months.

Some clinical trials, e.g., the German CML IV Study, have assessed doses of imatinib higher than the standard 400 mg daily to improve efficacy [1]. Of 422 patients randomized to the high-dose imatinib arm in the CML IV study, 68 reduced to imatinib 400 mg daily after achieving MMR [49]. Sixty-one of 68 remained in MMR after dose reduction, 5/68 transiently lost MMR, but regained this while continuing on imatinib 400 mg daily. Only 2/68 patients (3%) switched to a more potent TKI to regain MMR. These results demonstrate that reductions in treatment intensity for patients on high-dose imatinib (reduction from 800 mg to 400 mg) in patients in MMR are feasible and safe with the vast majority of patients maintaining MMR.

The information presented above provides evidence that dose reduction/interruption is safe and feasible. It has no effect on overall survival and minimal effects on molecular and cytogenetic responses. Importantly, dose reduction/interruption does improve side effect profiles and is a suitable method for managing many adverse events. However, it is unclear whether these strategies should be adopted more widely and not just for management of adverse events. Further prospective studies are required to evaluate this and also determine if there is a minimum effective dose of TKIs which should be considered prior to switching to an alternative TKI.

### Consideration of Dose Reduction for Patients in DMR

For optimally responding patients, TKI dose reduction may be considered for the management and prevention of adverse events, leading to improved quality-of-life. However, to date, these have been very limited prospective evaluation of this approach. There are two potential scenarios: (1) patients continuing long term on a reduced dose of TKI as “maintenance therapy” with a resultant improvement in tolerability and (2) patients reducing TKI dose prior to a TFR attempt.

In a small study, 43 patients in DMR on imatinib 400 mg daily for a median of 4.1 years had a dose reduction to 300 mg daily [50]. With a median follow-up of 1.6 years on imatinib 300 mg daily, only 2/43 patients had lost DMR, but maintained MMR, not patients lost MMR. Improvements in side effects were observed in 23/37 patients that reported side effects at the time of dose reduction.

The UK De-Escalation and Stopping Therapy with Imatinib, Nilotinib or sprYcel (DESTINY) [51••] clinical trial set out to explore the two potential scenarios above, prospectively. In addition, for the first time, DESTINY explored the feasibility and safety of dose reduction and treatment cessation in patients in stable MMR, and not necessarily in DMR. One hundred seventy-four patients who had been on TKI therapy for at least 3 years and in stable MMR (*n* = 49) or DMR (*n* = 125) for at least 12 months were recruited. Patients with prior documented resistance were excluded. Enrolled patients received 50% standard dose of TKI (IM 200 mg daily (*n* = 148), nilotinib 200 mg twice daily (*n* = 16), and dasatinib 50 mg daily (*n* = 8)) for the first 12 months, with monthly molecular monitoring. Patients maintaining MMR then attempted TFR and were followed for a further 24 months. During the 12 months of TKI reduction, molecular recurrence (defined as loss of MMR) occurred in 3/121 (2.5%) and 9/48 (18.8%) evaluable patients in the DMR and MMR groups, respectively. The recurrence rate was significantly lower in the DMR group (*P* = 0.0007). Importantly, all patients regained MMR within 4 months of restarting full-dose TKI. Interestingly, all recurrences on half-dose therapy occurred on imatinib and not a 2G-TKI, which may be a reflection of the higher potency of the 2G-TKIs. During the first 3 months of dose reduction, there was a significant improvement in side effects. In addition, there were substantial financial savings made from the TKI drug budget.

The UK DESTINY trial demonstrates that dose reduction is safe and feasible for patients in DMR. It is associated with the maintenance of molecular response in the vast majority of patients, especially if DMR has been achieved and maintained, as well as significant improvements in side effects and cost savings.

In the second phase of the DESTINY trial [52•], TFR after dose reduction was attempted for all patients remaining in MMR after 12 months of dose reduction. After a further 24 months of monitoring in TFR, the recurrence-free survival rate (defined as maintenance of MMR) was 72% in the DMR group and 36% in the MMR group. The only factor predictive of recurrence was treatment duration, and there was no association between recurrence and age, gender, performance status, or prior therapies. There were 2 non-CML-related deaths and no progressions to advanced phase. All episodes of molecular recurrence re-achieved MMR within 5 months. Again, there was a further improvement in symptom burden during the first few months of TFR. The period of dose reduction did not prevent symptoms of the TKI withdrawal syndrome which occurred in 21% of patients [53].

The excellent successful TFR rate of 72% in the DESTINY DMR group raises the controversial question as to whether dose reduction prior to stopping in some way improves the outcome. The TFR rate in the DESTINY DMR group is substantially better than that of the much bigger EUROSKI clinical trial [54•], which at 2 years had a recurrence-free survival rate of 50%; both studies had almost identical inclusion criteria. The potential mechanisms behind this are unclear, but effects on CML stem cells, the immune system and improved compliance during the dose-reduction phase may all contribute. Further studies are now warranted to define the optimal dose-reduction strategies, not only for patients wishing to continue on long-term reduced dose TKI maintenance, but also for patients in DMR considering TFR. While dose reduction for side effects for patients in MMR is safe and feasible, provided adequate monitoring is available, TFR attempts should be reserved for those patients in DMR, except in exceptional circumstances.

## Conclusions and Future Directions

Over many years, multiple clinical trials across all TKIs have demonstrated the importance of dose optimization via dose modifications. The two main goals of this approach are to maintain the excellent efficacy of TKIs while reducing side effects. Side effects may be persistent and low grade, such as the chronic fatigue and fluid retention associated with imatinib, where persistence over many years impacts quality-of-life, more serious such as pleural effusion associated with dasatinib, or life threatening, e.g., the increased ATEs seen with nilotinib and ponatinib.

Dose modifications may be considered at all stages of the patient’s treatment journey. Evidence is accruing that dose modifications are safe and feasible throughout treatment and are an important consideration for the prevention and management of side effects, improving adherence and reducing treatment interruptions. Dose modifications may be particularly useful in the elderly or those with multiple comorbidities.

A list of currently recruiting dose-optimization/modification studies is shown in Table 2. Novel, prospective clinical trials are required to explore dose optimization in CP-CML, from newly diagnosed patients through those experiencing resistance or intolerance to those wishing to attempt TFR. These important studies will lead to further improvements in quality-of-life and outcome for CP-CML patients.

**Table 2:** Current and ongoing TKI dose-optimization/modification clinical trials in CML list on the www.clinicaltrials.gov website (Accessed on February 28, 2019)

Trial title	TKI(s)	Patient population	Primary endpoint	NCT number
Effect of pharmacogenetics on imatinib plasma level and response	Imatinib	CML on imatinib for at least 12 months (*n* = 100)	MMR	NCT03262974
Gleevec as maintenance therapy after cytogenetic response with nilotinib in newly diagnosed chronic myelogenous leukemia	Nilotinib Imatinib	Newly diagnosed CP-CML (*n* = 25)	Maintenance of CCyR on imatinib after nilotinib induction	NCT01316250
The efficacy and safety of induction-maintenance protocol for patients with chronic myelogenous leukemia	Dasatinib Nilotinib Imatinib	Newly diagnosed CP-CML (*n* = 15)	Maintenance of molecular progression-free survival	NCT03241199
Optimization of TKIs treatment and quality-of-life in Ph + CML patients ≥ 60 years in deep molecular response	Dasatinib Nilotinib Imatinib	CP-CML aged ≥ 60 in MMR/MR4.0 (*n* = 502)	Changes in quality-of-life	NCT02326311
KISS study: Kinase Inhibition with Sprycel Start (KISS)	Dasatinib Imatinib	Newly diagnosed CP-CML (*n* = 100)	Maintenance of MMR 2 years after switch to imatinib	NCT03193281
Low-dose dasatinib as first-line treatment for chronic myeloid leukemia	Dasatinib	Newly diagnosed CP-CML (*n* = 12)	Molecular response at 6 months	NCT03216070
Low-dose dasatinib (50 mg daily) as first-line treatment for newly diagnosed chronic phase chronic myeloid leukemia	Dasatinib	Newly diagnosed CP-CML (*n* = 100)	MMR at 12 months	NCT03625388
Dasatinib Holiday for Improved Tolerability (DasaHIT)	Dasatinib	Newly diagnosed CP-CML and resistance/intolerance to first-line TKI in CP-CML (*n* = 306)	Cumulative toxicity score and MMR	NCT02890784
Phase II study testing the tolerability of bosutinib in chronic phase CML patients (BODO)	Bosutinib	Resistance/intolerance to first-line TKI in CP-CML (*n* = 127)	Rate of GI-toxicity within the first 6 months of treatment	NCT03205267
Bosutinib dose-optimization study in chronic myeloid leukemia (CML)	Bosutinib	Resistance/intolerance to first-line TKI in CP-CML (*n* = 42)	Rate of MCyR	NCT02906696
Bosutinib in Elderly Chronic Myeloid Leukemia (BEST)	Bosutinib	Resistance/intolerance to first-line TKI in CP-CML (*n* = 65)	Rate of MMR at 12 months	NCT02810990
Activity and risk profile of ponatinib in chronic phase patients with chronic myeloid leukemia resistant to imatinib	Ponatinib	Second-line after failure of first-line imatinib therapy (*n* = 78)	Rate of MCyR at 12 months	NCT02398825
Ponatinib in participants with resistant chronic phase chronic myeloid leukemia (CP-CML) to characterize the efficacy and safety of a range of doses (OPTIC)	Ponatinib	CP-CML with failure of at least two prior TKIs (*n* = 276)	Rate of BCR-ABL:ABL ratio of ≤ 1% at 12 months	NCT02467270

*CCyR*, complete cytogenetic response; *CP-CML*, chronic phase chronic myeloid leukemia; *MCyR*, major cytogenetic response; *MMR*, major molecular remission; *MR4.0*, molecular remission 4.0
